# Nerve Conduction Study and Functional Assessment After Upper Extremity Macroreplantation

**DOI:** 10.3390/jcm14248818

**Published:** 2025-12-12

**Authors:** Sławomir Kroczka, Magdalena Jaworek, Marta Lecznar-Piotrowska, Małgorzata Steczkowska, Anna Grela, Aleksandra Gergont

**Affiliations:** 1 Department of Child and Adolescent Neurology, Jagiellonian University Medical College, 30-663 Krakow, Poland; slawomir.kroczka@uj.edu.pl (S.K.); anna.grela@uj.edu.pl (A.G.); ola.gergont@uj.edu.pl (A.G.); 2 Malopolskie Center of Hand Rehabilitation (MCRR), Ludwik Rydygier Specialistic Hospital, 31-826 Krakow, Poland; ccr-sekretariat@rydygier.krakow.pl

**Keywords:** upper limb replantation, nerve conduction study, functional assessment, rehabilitation

## Abstract

**Objectives:** The recovery of arm function after macroreplantation is influenced by various factors. The aim of this study was to present the results of functional rehabilitation outcome after replantation of an upper extremity. Moreover, we assessed nerve conduction validity in the process of monitoring the return of manual functions. **Methods:** The study was performed in a group of seven patients after upper extremity macroreplantation and rehabilitation. Assessments included measuring hand/arm function loss using Swanson’s method, range of motion, muscle strength, sensation, and manipulation dexterity through the NHPT (Nine-Hole Peg Test). The nerve conduction study measured response amplitude, conduction speed, and distal latency. **Results:** The average loss of function of the hand diminished from 63.6% to 49.18%. Significant improvement in global pressure was achieved. In the functional capacity test (NHPT), the average time of the test was improved. The final nerve conduction study demonstrated improvements in motor and sensory conduction parameters. A correlation between improvement in conduction parameters in sensory fibers and sensation in the two-point discrimination test was found. Increased potential amplitude in motor fibers of the examined nerves correlated with a decrease in loss of function of the arm. **Conclusions:** Functional assessment and tailored rehabilitation strategies would maximize recovery potential after macroreplantation. Nerve conduction remains a crucial tool in monitoring the progress of manual skills after months of rehabilitation. Our findings highlight the importance of long-term follow-up of these patients.

## 1. Introduction

The first successful human limb replantation procedures were conducted in the early 1960s. In recent years, the criteria for qualifying patients for replantation have broadened [1,2,3,4,5]. Currently, replantation for injuries previously considered disqualifying for surgery, e.g., avulsion amputation and multiple-level amputation, is often successfully achieved [6,7,8,9,10,11,12].

Despite the developments in microsurgery, replantation procedures involving the upper extremity still remain highly challenging [5,6]. Proper preoperative protection and transportation of the severed limb [13], which should not exceed 60 min, and limiting the period of primary ischemia to 6 h [2,3] are necessary; these are achieved by applying vascular catheters or bridges [2,6,14]. An important challenge for the long-term care of patients following replantation is their disproportionately high expectations regarding the possibility of regaining full manual dexterity in the future.

Surgical treatment outcomes in the case of arm amputation do not always result in a return to full dexterity [5,6], especially in regard to high amputations at the level of the shoulder, forearm, or wrist. According to several authors, replantation sometimes offers the patient fewer advantages than simple amputation [15].

Urso-Baiarda [16] demonstrated that as many as 40% of patients with severe arm injuries did not return to their previous professions, whereas in the case of the remaining patients, the treatment process and returning to a prior professional activity usually took a long time, often more than a year. Moreover, a large group of the patients required secondary reconstructive surgeries in the form of tenolysis of the tendons, neurolysis of the nerves, or arthrolysis of the joints [10,17,18,19,20]; such surgeries significantly extended the course of treatment, increased its costs, and aggravated psychological problems.

The therapeutic process together with amputation in the area of the upper limb proceeds in stages, and the return of function in the arm depends on the skill and cooperation of many specialists: a team of surgeons, neurologists, psychiatrists, and rehabilitation specialists [21,22,23,24]. In order to achieve good treatment results, it is also important to monitor the progress of therapy, which enables doctors to make decisions faster when it is necessary to perform secondary reconstructive surgery and has a significant positive impact on the patient’s psyche and involvement in the healing process.

The aim of the study was to present the results of functional outcome after replantation of an upper extremity. Moreover, we assessed the nerve conduction study’s validity in the process of monitoring the return of manual functions and its influence on further treatment (e.g., secondary reconstructions, rehabilitation). Materials and methods are described in detail. Results are graphically presented, discussed, and summarized to draw conclusions.

## 2. Materials and Methods

The study group consisted of 7 patients (5 men and 2 women with an average age of 50.7 years ± 20.7 years) following macroreplantation of the upper extremity after trauma injury. Two patients’ injuries were at the level of the metacarpus, two at the level of the wrist, and three at the level of the forearm. All patients underwent rehabilitation at the Małopolskie Center of Hand Rehabilitation (MCRR) in the Ludwik Rydygier Specialistic Hospital. Five patients had surgery on the right upper extremity, and two patients had surgery on the left upper extremity; all were right-handed. The mechanisms of the injuries were as follows: a circular or mechanical saw in five cases (71.5%), and guillotine trauma from being cut by a machete or sheet of metal in two cases (28.5%).

Rehabilitation was initiated 4 months on average from the macroreplantation procedure and was related to the type of injury, the level of damage, and the process of healing. Rehabilitation was conducted in 6-week-long sessions, 5 days a week, with 3-to-4-week breaks between sessions. On average, the test group underwent 3 rounds of rehabilitation lasting 6 weeks (i.e., 18 weeks). The shortest period was 12 weeks for a person with an injury at the level of the metacarpus, and the longest was 24 weeks in the case of 2 patients.

Rehabilitation was conducted based on exercises using the pegboard system and physiotherapy treatments according to protocols of exercises published in references [22,23,24,25]. In order to stimulate sensation and improve arm function, mirror therapy was also used with each patient, in addition to exercises in tactile memory by immersing the arm in various plastic masses, grains of wheat, and mustard seeds, as well as visual biofeedback based on computer programs. An assessment of the improvement of arm function was conducted based on clinical and electrophysiological tests. In the clinical test, the assessment included the following:

1. Muscle power as measured by a Jamar dynamometer [26] (measurements were assessed in the spacing of the dynamometer on the second level G2 and on the fourth level G4);
2. Sensation using the test of two-point discrimination with a discriminator [26,27] (the test was conducted on the radial and ulnar side of all fingers);
3. Manipulative dexterity test—NHPT (Nine-Hole Peg Test) [26];
4. Hand function was calculated with the help of Swanson’s methodology, which uses previously tested goniometric measurements of active movements in order to assess the functions of the arm comprehensively, as well as the results of sensation tests [27].

In the nerve conduction study (NCS), the assessment involved: latency (Lat) and distal latency (dLat), amplitude (A), and the speed of conduction (CV) of a complex motor potential (CMAP) as well as sensory potential (SNAP). For each patient, the median, radial, and ulnar nerves were examined. In regard to examining the sensory nerves, orthodromic stimulation was used while taking into consideration conduction from the index finger with stimulation of the fibers of the median nerve and to the little finger with stimulation of the ulnar nerve.

Clinical tests were conducted at the beginning and end of each six-week treatment session, and nerve conduction study tests were conducted every three months on average. In the final assessment, the results of the first test in the first treatment session as well as the last one in the last treatment session were considered—four months and twelve months after macroreplantation.

The results were compared with those obtained in the control group, which comprised 7 healthy patients in the same age range (two females, 5 males, mean 50.7 years ± 20.7 years). The clinical characteristics of the patients and control enrolled in the study are shown in Table 1.

Statistical analysis was performed using the statistical package R version 3.2.4. (*p*-value < 0.05 was considered statistically significant). The test of statistical significance was performed using the Wilcoxon single-rank test. To evaluate correlations between the clinical tests and the electrophysiological assessments, the Yule phi coefficient was used because of the group’s small number. We compared the parameters of conduction in the fibers of each motor and sensory nerve with loss of hand function and grip strength globally in levels G2 and G4. The correlation coefficient was determined by the Spearman method.

## 3. Results

**Figure 1:**
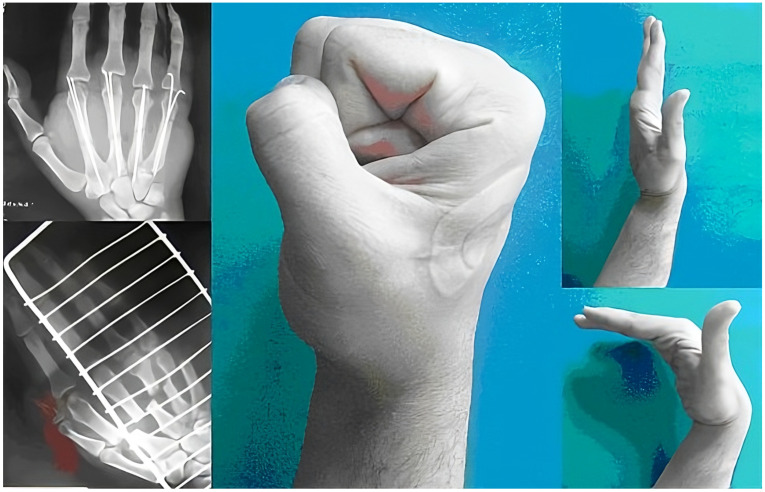
Post-replantation functional assessment (published with the patient’s permission).

In the studied group, the average loss of hand function measured with the Swanson method was 63.6% at the beginning of treatment, whereas it was 49.18% in the final test following rehabilitation 12 months from the date of the injury. The greatest improvement in hand function was seen in the patients who had experienced guillotine trauma at the level of the forearm, at 26%, and at the level of the wrist, at 13.5%. (Figure 1).

In an examination of the power of global grip measured in kilograms with a Jamar dynamometer on the levels of G2 and G4 in the initial study, similar values were achieved. Following rehabilitation, a significant improvement in global grip was achieved, both in G2 grip (from mean 0.86 kg in the initial examination to mean 3.93 kg in the final examination) and in G4 grip (from mean 0.91 kg to 7.33 kg). In group G2, the Wilcoxon signed-rank test revealed a statistically significant increase in global grip strength between the initial and final examinations (*p* = 0.001). In group G4, the improvement was more pronounced and also highly significant (*p* = 0.0003). Furthermore, the Wilcoxon rank-sum test comparing the two groups demonstrated that, at the final examination, global grip strength in G4 was significantly higher than in G2 (*p* = 0.0002).

**Figure 2:**
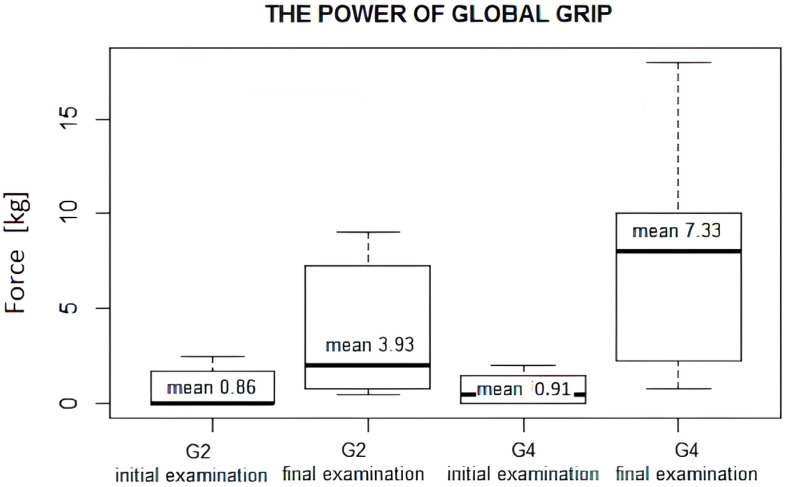
The power of global grip G2 and G4 mean values at 4 and 12 months after macroreplantation/initial and final examination measurements. Initial examination—4 months after macroreplantation. Final examination—12 months after macroreplantation.

Improvement in grip strength G2 and G4 and reduction in loss of hand function were statistically significant (Figure 2).

By contrast, after conversion to the percent of the strength of a healthy limb, an improvement in the power of global grip was achieved after approximately 12 months of treatment in the G2 (10%) and G4 grip (23%).

In the functional capacity test (NHPT), significant improvement was achieved in the final test (from 451.75 s to 67 s). However, it must be noted that in the study group, three people were unable to perform the test at the beginning and at the end of the treatment; this was related to coexisting injuries. The results are presented in Table 2.

Protective sensation was found in five cases (71%), including three people (42.8%) with sensation at the level of 10 mm; two people (28.5%) scored 0 points—no sensation—in the final test during the two-point discrimination test.

In the initial nerve conduction study performed 4 months after injury, all patients displayed no response of motor and sensory fibers to supramaximal stimulus. Four to five months after injury, the first motor response was observed as residual amplitude, significantly slowed conduction, and highly extended distal latency in the radial nerve in three patients and the median and ulnar nerves in one person (with replantation at the level of the metacarpus). Clear CMAP appeared approximately 7 to 10 months after injury but still with lower parameters of conduction. After 12 months, proper parameters of conduction occurred in motor fibers of the radial nerve in two patients and the median nerve in one patient (injury at the level of the metacarpus). In sensory fibers during orthodromic stimulation, the first responses occurred within month 6 to month 7 and were often vestigial. Only in two cases were correct parameters of conduction in sensory fibers of the examined nerves obtained.

The final conduction study demonstrated an improvement in the parameters of motor conduction of the median, ulnar, and radial nerves as well as in sensory fibers of the median and ulnar nerves. The greatest improvement was observed in increasing the amplitude and reducing the distal latency. The reduction in latency in sensory nerve fibers of the median and ulnar nerves was statistically significant, whereas sensory conduction speed in the ulnar and radial nerves slowed down slightly. Motor and sensory conduction parameters were compared in the injured limb in each patient before and 12 months after rehabilitation (Table 3).

Based on the Yule phi calculation, positive changes in respective parameters in the motor and sensory units corresponded to positive changes in clinical parameters. A weaker but also positive correlation was observed in the case of conduction velocity in the sensory fibers. In addition, based on the Spearman correlation coefficient, it was shown that improvement in the loss of hand function, as measured by Swanson, corresponded to an increase in muscle strength at G2 and G4. A correlation has also been shown in the median nerve. The amplitude increases in the motor fibers correlated with an increase in muscle strength for G2 and G4. An increase in the amplitude of the sensory fibers corresponded with a decrease in the loss of hand function, while shortening the latency in the sensory fibers of the median nerve correlated with an improvement in sensation in the two-point discrimination test. In other nerves, a marked tendency for an increase in amplitude in motor fibers correlated with a decrease in loss of function of the hand, but it was not statistically significant. There was no correlation between the parameters of conduction in the sensory fibers of the studied nerves and increased muscle strength in grip on G2 and G4 (Table 4, Table 5, Table 6 and Table 7).

During the observation, five of seven of the patients (71.4%) underwent repeated reconstruction procedures; two people who were subjected to secondary surgeries were injured at the level of the metacarpus, and three were injured more proximally. All of them had at least three reconstructive procedures. All underwent the procedures of tenolysis of flexor tendons and neurolysis of nerves, and the remaining surgical procedures included z-plasty of the skin, muscle transfers, and arthroplasty of the joints.

## 4. Discussion

Megareplantation of an upper extremity is a challenging surgical procedure. The patient should be aware of long-term rehabilitation, and the potential lack of a return to full mobility of the upper limb [21,22,28,29,30,31,32].

The task of the entire treatment team is to achieve a scope of passive and active movement of the joints, muscle power, and functional dexterity of the upper extremity that is as close to normal as possible. In the references, several authors based their assessment of improvements in arm function on the DASH questionnaire [17,33]; others based it on the assessment of improvements in muscle power measured in kilograms; and others based it on the percentage of strength of the healthy upper extremity. The occurrence of the function of thumb opposition and the ability to make a fist were also taken into consideration. Research considered the degree of patient satisfaction and the percentage of patients returning to professional work, in addition to sensation in the two-point discrimination test. According to our knowledge, only a small number of authors also considered the results of the EMG test. In the group analyzed in this paper, clinical evaluations were combined with the results of electrophysiological tests.

In our study, comparable results were achieved when examining muscle power in grip for G2 and G4 in the initial test. Also in the references, most patients after replantation at the level of the wrist and distal part of the forearm achieved an improvement in the force of global grip (most commonly presented at the level of G2). According to Sturm et al. [29], this improvement after 13 months of treatment was 3–4 kg; according to Jabłecki et al. [7], after 3.5 years of treatment, it was 5.4 kg; Gulgonen and Ozer [31] described the return of global grip power as 34% of the power of an intact healthy limb after 5 years of treatment; and according to Patel, the force of global grip is only 9% of the power of the healthy upper extremity after 4 years of treatment following an injury [28]. The differences in the data obtained may result not only from various times of the performed checkups (13 months to 5 years) but also from different methodologies of the conducted research and the level of the injury. When we compare the results of muscle power of our patients to the results presented in the references, we can conclude that they do not differ from the results of the above-mentioned authors. The differences in the power of grip on G2 and G4 in the initial and final tests may be explained by the extent of the injury and the level of damage. For patients with a high amputation, apart from flexor tendons, vessels and nerves were damaged, including the ulnar nerve that provides movement for the inner muscles of the arm. The proper functioning of the inner-arm muscles has a significant influence on the grip power G2, whereas external flexors of the fingers greatly influence grip G4.

The improvement in the manipulative dexterity of the hand as measured with the NHTP test from the 7th min to 1st min was not statistically significant. This was probably connected to the low number of individuals studied (three people were unable to perform the test at the beginning and at the end of the treatment due to coexisting injuries), and for the remaining four people, the results after treatment were very good. The NHPT test is closely connected to precision grip, and people who did not perform this test had an injury at the level of the forearm, and restoration of function influenced global actions to a greater degree than precise function actions. Moreover, these patients were waiting for further reconstruction procedures.

After replantation at the level of the wrist and forearm, the values obtained by patients in retrospective tests after 5–6 years, according to various authors, amounted to an average of 10.6 mm or 20 mm [34,35]. Aligning with the findings of Gulgonen et al. [31] and Kraup et al. [36], all patients in the study group achieved sensation at least at the level of protective sensation.

It is difficult to compare the improvement in arm function in the studied group with improvements in arm function presented in the references. Moreover, Swanson’s method, which was used to calculate loss of arm function in our study, was more complex (it took into account combined measurements of mobility, sensation, and level of amputation) compared to the measurements of other authors. Still, in alignment with most of the authors [7,10,17,21,28,30,33,34,35,37], we may state that, in regard to macroreplantation, the function of the limb is returned to a satisfactory level, although the replanted arm is never as fully functional as the healthy limb.

NCS performed in our study group 12 months after replantation revealed significantly improved parameters of conduction in motor fibers of the median, ulnar, and radial nerves as well as in the sensory fibers of the median nerve, especially in terms of growth of amplitude and reduction in latency. However, in the sensory fibers of the ulnar and radial nerves, a decrease in the velocity of conduction was noted together with an increase in amplitude and a reduction in distal latency. Moreover, correct parameters of conduction after approximately 12 months of treatment were obtained in three cases. Krarup et al. [36], similarly to our study, achieved the greatest improvement in the area of increase in amplitude. By contrast, in sensory fibers, no response was obtained, even in arms with good sensory function [35,37]. A comparison of our results and the results in the references demonstrated that an increase in the amplitude of motor fibers is a parameter that most closely correlates to an improvement in arm function. An improvement in the parameters of conduction in sensory fibers is noted later than in motor fibers [35]. Worse parameters of the sensory conduction speed may be connected to the primary reconstruction procedure (a shorter time required for reconstruction of sensory fibers until 4 months from the injury in connection with atrophy of sensory cells compared to reconstruction of motor fibers until about a year), and with the presence of extensive scars and secondary changes that draw surrounding tissues, including nerves, into the scarring process. Perhaps it was also connected to a greater focus on improving more damaged structures (the radial nerve in our own research was damaged to the smallest degree in most patients in the initial test) and secondary reconstruction, mainly within the median and radial nerves in the analysis of the degree of denervation and reinnervation of the researched muscles.

A combination of clinical and electrophysiological methods provides ground for the reliable assessment of improvements in arm function. According to Sabapathy et al. [32], secondary procedures are difficult because they carry the risk of damaging important neuromuscular structures supplied during replantation, the location and position of which different anatomically after the primary procedure.

Finally, it must be noted that the tests of the analyzed group of patients in the present paper were conducted in the early stage (up to 12 months after the injury) compared to tests described in the cited research of other authors. The results would probably be much better during long-term observation. After observation lasting one year and rehabilitation, the patients regained function of their limb to differing degrees. Most were able to perform simple daily activities, but there are also cases where the results of treatment were very good, depending largely on the patient’s motivation and well-conducted rehabilitation. Undoubtedly, the limitation of this study is related to the low number of participants in the research group.

## 5. Conclusions

In conclusion, the restoration of motor function after megareplantation is a complex process depending on various factors. The average loss of function of the hand diminished from 63.6% to 49.18%. Significant improvement in global pressure was achieved. In the functional capacity test, the average time of the test was improved. The final electroneurography demonstrated improvements in motor and sensory conduction parameters, correlated with sensation in two-point discrimination. Increased potential amplitude in motor fibers of the examined nerves correlated with a decrease in loss of function of the arm. Our findings highlight the importance of long-term follow-up of the patients and correlations between clinical outcomes and electrophysiological data after months of rehabilitation. Functional assessment and tailored rehabilitation strategies would maximize recovery potential. Moreover, NCS seems to be a crucial tool in monitoring the progress of arm function after megareplantation. Achievement of a satisfactory clinical effect is based on the cooperation of the therapeutic team.

## Figures and Tables

**Table 1:** Baseline characteristics of the patients enrolled in the study.

Measurements		Study Group n = 7		Control Group n = 7	
**Age, [years, mean ± SD]**		50.7 ± 20.7		50.7 ± 20.7	
**Gender, [Male/n, %]**		5/7	71%	5/7	71%
**[Female/n, %]**		2/7	29%	2/7	29%
**Localization of arm injury**	**Metacarpus**	2/7	29%		
	**Wrist**	2/7	29%		
	**Forearm**	3/7	42%		
**Mechanism of arm injury**	**Guillotine**	2/7	29%		
	**Saw**	5/7	71%		

SD—standard deviation.

**Table 2:** Functional assessment of the patients enrolled in the study.

Measurements	Study Group n = 7	Control Group n = 7	*p*-Value Study vs. Control
Power of global grip G2 I [kg]	0.86 ± 1.1	39 ± 13.7	*p* = 0.0015 *
Power of global grip G4 I [kg]	0.91 ± 0.9	31.7 ± 12.78	*p* = 0.0015 *
Power of global grip G2—II [kg]	3.93 ± 3.76	39 ± 13.7	*p* = 0.0017 *
Power of global grip G4—II [kg]	7.33 ± 6.11	31.7 ± 12.78	*p* = 0.0016 *
NHPT I [s]	178.28 ± 281.15	25.85 ± 3.62	*p* = 0.0220 *
NHPT II [s]	44.57 ± 50.42	25.85 ± 3.62	*p* = 0.6110

I—first examination—four months after macroreplantation; II—final examination—twelve months after macroreplantation; NHPT—Nine-Hole Peg Test; * statistically significant.

**Table 3:** Nerve conduction study measurements (mean ± SD) at 4 (I) and 12 (II) months after macroreplantation.

Sensory Fibers	Latency [ms]		Amplitude [mV]		Conduction Speed [m/s]	
	I	II	I	II	I	II
Median nerve	20.53 ± 21.09	4.38 ± 1.40	1.27 ± 1.52	2.83 ± 2.49	28.42 ± 27.54	45.34 ± 16.15
Ulnar nerve	15.35 ± 18.98	3.85 ± 2.22	3.25 ± 5.21	4.37 ± 5.21	33.48 ± 27.82	33.28 ± 19.72
Radial nerve	9.42 ± 14.93	4.20 ± 1.60	6.98 ± 8.82	10.21 ± 12.87	41.32 ± 25.81	36.58 ± 22.92
**Motor Fibers**						
Median nerve	17.72 ± 17.20	5.34 ± 2.3	2.25 ± 1.94	2.62 ± 1.51	26.98 ± 10.75	42.51 ± 10.36
Ulnar nerve	13.83 ± 10.9	4.04 ± 1.01	2.35 ± 1.23	3.74 ± 1.44	34.77 ± 12.21	47.77 ± 8.39
Radial nerve	8.35 ± 6.51	2.08 ± 1.01	2.18 ± 1.02	3.49 ± 1.07	46.14 ± 12.32	61.92 ± 9.54

**Table 4:** Percentage ratio of nerve conduction study measurements between the study group and healthy controls at 4 (I) and 12 (II) months after macroreplantation.

Sensory Fibers	Latency		Amplitude		Conduction Speed	
	I	II	I	II	I	II
Median nerve	14%	67%	11%	26%	42%	68%
Ulnar nerve	17.5%	70%	33%	44.4%	48.6%	48.3%
Radial nerve	25%	56%	29%	43%	56%	50%
**Motor Fibers**						
Median nerve	19%	64%	37%	44%	50%	80%
Ulnar nerve	19.4%	67%	31%	50.5%	56%	77%
Radial nerve	31%	70%	44%	80.4%	60%	71%

**Table 5:** Spearman’s rank correlation (ρ, 95% confidence intervals) of sensory and motor nerve conduction parameters with power of global grip G2 and G4.

	G2			G4		
Sensory Fibers	Latency ρ, CI	Amplitude ρ, CI	Conduction Speed ρ, CI	Latency ρ, CI	Amplitude ρ, CI	Conduction Speed ρ, CI
Median nerve	−0.03 (−0.71; 0.69)	−0.10 (−0.81; 0.70)	0.20 (−0.60; 0.87)	0.07 (−0.72; 0.74)	−0.60 (−0.91; 0.23)	−0.07 (−0.79; 0.71)
Ulnar nerve	−0.20 (−0.90; 0.69)	−0.14 (−0.82; 0.70)	0.03 (−0.50; 0.86)	0.10 (−0.70; 0.81)	0(−0.71; 0.73)	−0.30 (−0.91; 0.64)
Radial nerve	−0.20 (−0.91; 0.65)	0.30 (−0.65; 0.91)	−0.07 (−0.79; 0.72)	−0.40 (−0.92; 0.53)	−0.1 (−0.80; 0.71)	0.30 (−0.67; 0.90)
**Motor Fibers**						
Median nerve	−0.20 (−0.90; 0.61)	0.56 * **(−0.1; 0.82)**	0.03 (−0.52; 0.83)	−0.10 (−0.73; 0.82)	0.79 * **(0.30; 0.95)**	−0.35 (−0.89; 0.57)
Ulnar nerve	−0.59 * **(−0.81; 0.09)**	0.18 (−0.53; 0.82)	0.20 (−0.91; 0.70)	−0.25 (−0.89; 0.73)	−0.11 (−0.79; 0.69)	0.17 (−0.56; 0.80)
Radial nerve	−0.10 (−0.82; 0.69)	0.45 (−0.46; 0.91)	0.44 (−0.46; 0.92)	−0.50 * **(−0.02; 0.72)**	0.32 (−0.49; 0.86)	−032 (−0.87; 0.50)

ρ—Spearman’s rank correlation coefficient; CI—95% confidence interval; * correlation coefficient.

**Table 6:** Spearman’s rank correlation (ρ, 95% confidence intervals) of sensory and motor nerve conduction parameters with the percentage loss of arm function.

Sensory Fibers	Latency ρ, CI	Amplitude ρ, CI	Conduction Speed ρ, CI
Median nerve	−0.20 (−0.81; 0.59)	0.60 * **(0.15; 0.81)**	0.30 (−0.51; 0.80)
Ulnar nerve	−0.07 (−0.69; 0.71)	0.30 (−0.52; 0.78)	0.07 (−0.71; 0.81)
Radial nerve	0.70 * **(0.15; 0.93)**	−0.14 (−0.72; 0.68)	−0.21 (−0.83; 0.57)
**Motor Fibers**			
Median nerve	−0.30 (−0.72; 0.49)	0.43 (−0.21; 0.91)	0.30 (−0.54; 0.83)
Ulnar nerve	0.03 (−0.75; 0.79)	0.47 (−0.21; 0.93)	0.42 (−0.33; 0.92)
Radial nerve	0.42 (−0.25; 0.89)	0.25 (−0.59; 0.88)	0(−0.76; 0.74)

ρ—Spearman’s rank correlation coefficient; CI—95% confidence interval; * correlation coefficient.

**Table 7:** Spearman’s rank correlation (ρ, 95% confidence intervals) of sensory nerve conduction parameters with two-point discrimination test.

Sensory Fibers		Latency ρ, CI	Amplitude ρ, CI	Conduction Speed ρ, CI
Median nerve	Radial side	0.6 * **(0.05; 0.89)**	0.3 (−0.49; 0.91)	0.4 (−0.21; 0.90)
	Ulnar side	0.8 * **(0.21; 0.94)**	0.2 (−0.61; 0.79)	0.64 * **(0.11; 0.85)**
Ulnar nerve	Radial side	0.2 (−0.73; 0.85)	0.07 (−0.76; 0.81)	−0.63 * **(−0.80; −0.14)**
	Ulnar side	0.1 (−0.69; 0.78)	0.07 (−0.73; 0.82)	−0.67 * **(−0.83; −0.25)**
Radial nerve	Radial side	0.14 (−0.65; 0.84)	0.25 (−0.59; 0.89)	−0.59 * **(−0.82; −0.05)**
	Ulnar side	0.15 (−0.74; 0.68)	0.4 (−0.49; 0.93)	−0.6 * **(−0.91; 0.00)**

ρ—Spearman’s rank correlation coefficient; CI—95% confidence interval; * correlation coefficient.

## Data Availability

The data presented in this study is available on request from the corresponding author.

## Abbreviations

A	amplitude
CI	confidence interval
CMAP	Compound Muscle Action Potential
dLat	distal latency
Lat	latency
NCS	Nerve Conduction Study
NHPT	Nine-Hole Peg Test
SNAP	Sensory Nerve Action Potential
